# Extrudate *versus* Powder Silica Alumina as Support for Re_2_O_7_ Catalyst in the Metathesis of Seed Oil-Derivatives – A Comparison

**DOI:** 10.3390/ijms10010213

**Published:** 2009-01-08

**Authors:** Bassie B. Marvey

**Affiliations:** Department of Chemistry, North-West University P/Bag X2046 Mafikeng, South Africa. E-Mail: bassie.marvey@nwu.ac.za; Tel. +27-18-389-2527; Fax: +27-18-389-2052

**Keywords:** Olefin metathesis, extrudate silica alumina, powder silica alumina, 3wt% Re_2_O_7_, SiO_2_-Al_2_O_3_, SnBu_4_

## Abstract

Self- and cross-metathesis of fatty acid methyl esters (FAMEs) was investigated using a silica alumina supported Re_2_O_7_ catalyst. Although a 3 wt% Re_2_O_7_/SiO_2_-Al_2_O_3_/SnBu_4_ is already active for the metathesis of unsaturated FAMEs, the results have shown that particle size of silica alumina support has a profound influence on its activity and selectivity. Consequently, high substrate conversions coupled with improved product yields (for mono- and diesters) and reaction rates were obtained upon using powder, as opposed to extrudate silica alumina as the support material. Diesters are platform compounds for the synthesis of polymers and fragrances. In this paper a comparative outline of the influence of particle size of silica alumina (extrudate *versus* powder) on catalytic performance of a 3 wt% Re_2_O_7_/SiO_2_-Al_2_O_3_/SnBu_4_ for self- and cross-metathesis of FAMEs is made. Low surface area and diffusion constraints associated with extrudates were identified as some of the factors leading to low catalytic activity and selectivity.

## 1. Introduction

The first report on the heterogeneously catalysed metathesis of unsaturated esters was the work by Verkuijlen *et al*. [1] using Re_2_O_7_ supported on γ-Al_2_O_3_. Later on other support materials were found to also be effective, including silica alumina (SiO_2_-Al_2_O_3_) with varying Al_2_O_3_ content [2–5]. The use of SiO_2_-Al_2_O_3_ support on Re_2_O_7_ catalyst resulted in improved catalytic performance compared to γ-Al_2_O_3_ [2]. Furthermore, a SiO_2_-Al_2_O_3_ support with 24.3% Al_2_O_3_ content was the most active of all other SiO_2_-Al_2_O_3_ types used [2]. Hence for this study SiO_2_-Al_2_O_3_ with 24.3% Al_2_O_3_ was the support of choice. Silica alumina is characterized by Brönsted acidity which accounts for its pronounced activity.

**Scheme 1.**
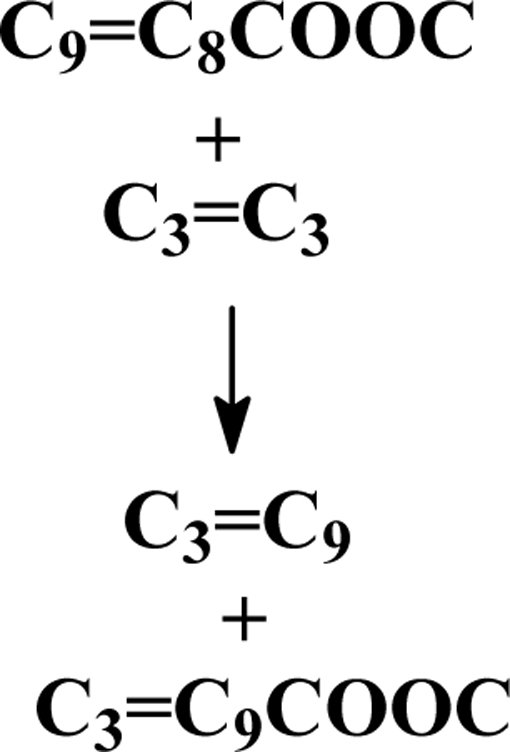
Cross-metathesis of methyl oleate with 3-hexene.

The metathesis of unsaturated fatty acid methyl esters (FAMEs) is an elegant method for the single-step synthesis of *n*-olefins, mono- and diesters of varying chain lengths and degrees of unsaturation [6–8]. Such products could find application as platform chemicals for the synthesis of niche market products. Monounsaturated fatty esters can, for example, be converted *via* a self-metathesis reaction into unsaturated diesters, which are interesting starting materials for the synthesis of cross-linked unsaturated polyesters and polyamides [7]. Through cross metathesis with short-chain alkenes, long-chain fatty esters can be shortened into detergent-range C_12_–C_14_ acid esters [9]. Cross-metathesis of methyl oleate with 3-hexene, for example, gives 3-dodecene and methyl 9-dodecenoate as illustrated in Scheme 1 [9]:

The abundance of unsaturated fatty acids and esters in animal fats and plant oils presents us with the opportunity to exploit these materials as renewable alternatives to petroleum-based feedstocks [10–14]. Direct transformation of unsaturated fats and oils by metathesis results in the formation of detergent-range alkenes and high molecular weight macromolecules with improved drying properties [7, 15]. Hence the metathesis of fatty oils provides a novel way of producing feedstocks from readily available and cheap raw materials.

The main challenge, however, is to find highly active, selective and stable catalysts that can withstand deactivation under a broad range of organic functional groups. Previously we reported the self- and cross-metathesis of sunflower oil-derived fatty esters using powder 3 wt% Re_2_O_7_/SiO_2_-Al_2_O_3_ catalyst [16]. In this paper a comparative outline of the influence of particle size of silica alumina (extrudate *versus* powder) on the activity and selectivity of a 3 wt% Re_2_O_7_/SiO_2_-Al_2_O_3_/SnBu_4_ for self- and cross-metathesis of FAMEs is presented. Previous related work by other authors focused primarily on the variation of alumina content in silica alumina in a single component substrate system, namely, methyl oleate [2, 5].

## 2. Materials and Methods

Hexanes (Aldrich) were dried by refluxing over sodium and were subsequently stored under an argon atmosphere. Methyl linoleate (Aldrich, 99%), methyl oleate (Aldrich, 99.0%) biodiesel and sunflower oil (Excella) were purified from peroxides by treatment with calcined silica alumina (SiO_2_-Al_2_O_3,_ Akzo-Nobel, 24.3% Al_2_O_3_, 347 m^2^·g^–1^) under N_2_ atmosphere. SiO_2_-Al_2_O_3_ was purchased in extrudate form from which powder silica alumina (125–180 μm particle diameter, 380 m^2^·g^–1^, 0.91 cm^3^·g^–1^ pore volume) was prepared. Biodiesel was prepared from sunflower oil by transesterification using methanol [5, 8]. Ammonium perrhenate (NH_4_ReO_4_, Fluka or Strem) and tetrabutyltin (SnBu_4_, Aldrich, 93%, were used as purchased. A 3 wt% Re_2_O_7_/SiO_2_-Al_2_O_3_ catalyst was prepared by pore volume impregnation of SiO_2_-Al_2_O_3_ (0.97 g) with an aqueous solution of NH_4_ReO_4_ (0.0332 g). The catalyst was dried at 120 °C followed by calcination in air at 550 °C for 3 h and subsequently under N_2_ for 1 h. To 3 wt% Re_2_O_7_/SiO_2_-Al_2_O_3_ catalyst (0.2 g) in a glass reactor, solvent (hexanes, 2 mL) and the SnBu_4_ (5 μL) were added in that order, followed by the substrate (0.5 mL) after 2 min. of stirring. Samples were collected at regular time intervals with a syringe and were analysed by FID-GC equipped with a DB-1 fused silica capillary column and GC/MS as previously described [16].

## 3. Results and Discussion

### 3.1. Self-metathesis of methyl linoleate

**Figure 1.**
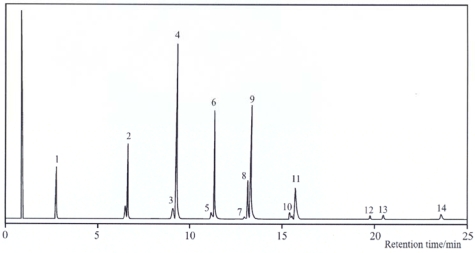
Gas chromatogram of the metathesis products of methyl linoleate: *n*-olefins (peaks 1–3, 5, 7), monoesters (peaks 4, 6, 8, 10, 12) and diesters (peaks 9, 11, 13, 14).

**Scheme 2.**
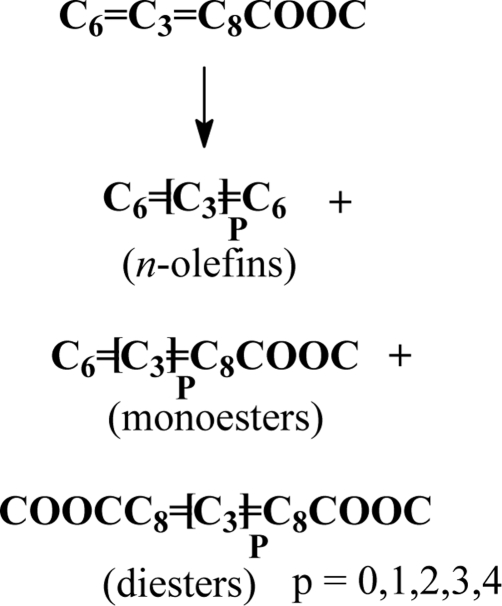
Self-metathesis of methyl linoleate.

Self-metathesis of methyl linoleate resulted in a mixture of *n*-olefins, monoesters and diesters (Scheme 2). Figure 1 shows a typical gas chromatogram for the products resulting from this self-metathesis, as well as its cross-metathesis with methyl oleate. Table 1 presents total metathesis and the yields for *n*-olefins, monoesters and diesters obtained with extrudate and powder silica alumina as support materials for a 3 wt% Re_2_O_7_ catalyst. Total metathesis represents the sum total of all product yields in mol% and serves as an indicator for metathesis activity. Upon using powder silica alumina, a total metathesis of 86.1% was obtained compared to 68.6% with extrudate silica alumina support. The yields obtained with powder silica alumina for *n*-olefins, monoesters (excluding the substate) and diesters were 28.1, 37.5 and 20.4%, compared to extrudate silica alumina with 28.5, 24.1 and 16% for *n*-olefins, monoesters and diesters, in that order. The use of powder silica alumina brought an improvement in mono- and diester yields by 35.7 and 21.6%, respectively. On the other hand, a slight decrease in *n*-olefins by 1.4% was obtained. A 1:2:1 statistical product distribution for the powdered catalyst is in agreement with the results previously reported in the literature [17–19].

The relatively low activity by extrudate 3 wt% Re_2_O_7_/SiO_2_-Al_2_O_3_ catalyst could be attributed to low surface area and diffusion constraints which have a negative bearing on catalyst-substrate contact, resulting consequently to lower product yields and reduced reaction rates. A similar observation was made by Sibeijn and Mol [5] with low alumina catalysts (13–15.3%) having different specific surface areas where the catalyst with high specific surface area gave the highest substrate conversion. A slight improvement in catalytic activity was, however, observed when extrudate silica alumina support was first pretreated by impregnating it with an aqueous solution of either 2 wt% PO_4_^3–^ or 2 wt% Cs^+^ ions. Pretreatment with 2 wt% PO_4_^3–^ or 2 wt% Cs^+^ ions led to an improved substrate conversion by 3.3 and 7.6%, respectively. These results were in agreement with those previously reported by du Plessis and coworkers for 1-octene [20]. However, Mol and Andreini [21] observed only an improvement in selectivity upon treatment of silica alumina with 2 wt% Cs^+^ ions in 1-octene metathesis.

### 3.2. Cross-metathesis of methyl linoleate with methyl oleate

Cross-metathesis of methyl oleate and methyl linoleate resulted in a mixture of *n*-olefins, monoesters and diesters. Table 2 presents the total metathesis and the yields obtained with extrudate and powder silica alumina as support materials. Total metathesis of 66.8% was obtained in the presence of extrudate silica alumina after 5 h, which represents a decrease in activity by 2.6% compared to self metathesis of methyl linoleate using the same type of silica alumina. On the other hand, total metathesis of 76.2% was attained in 3 h with powder silica alumina support, an improvement in activity by 14.1% relative to extrudate silica alumina supported catalyst. The yields obtained with powder silica alumina for *n*-olefins, monoesters and diesters were 23.4, 33.4 and 19.4% compared to extrudate silica alumina with 38.0, 22.8 and 6.0% for *n*-olefins, monoesters and diesters, in that order. This represents an improvement in mono- and diester yields by 46.5 and 223.3%, respectively. On the other hand, a decrease in *n*-olefins by 38.4% was observed. Furthemore, higher molecular weight olefins (≥ C_21_), monoesters (≥ C_25_) and diesters (≥ C_26_) were not observed upon using extrudate 3 wt% Re_2_O_7_/SiO_2_-Al_2_O_3_/SnBu_4_ catalyst, probably as a result of a relatively low substrate conversion. These products were, however, observed with a powdered support.

In spite of the differences in catalytic activity, both powder and extrudate silica alumina supported Re_2_O_7_ catalysts showed preferential selectivity for products with fewer carbon-carbon double bonds. For that reason selectivity decreased in the order: monoenes > dienes > trienes > tetraenes. It follows, therefore, that higher yields obtained were for the compounds containing fewer carbon-carbon double bonds than was the other way round.

### 3.3. Metathesis of FAMEs derived from sunflower oil

While previously we reported the metathesis of FAMEs from sunflower oil using a powdered 3wt% Re_2_O_7_/SiO_2_-Al_2_O_3_/SnBu_4_ catalytic system [16], we now report the metathesis of the same using extrudate silica alumina supported 3 wt% Re_2_O_7_/SiO_2_-Al_2_O_3_/SnBu_4_ catalyst. The substrate mixture was composed of palmitate (7.3%), stearate (6%), oleate (20.6%), linoleate (65.1%), arachidate (0.3%), and behenate (0.7%). Table 3 compares the total metathesis and the yields obtained with extrudate and powder silica alumina supports. After 3 h reaction time, the total metathesis was 72.2% with powder silica alumina supported Re_2_O_7_ catalyst compared to 66.7% after 5 h with extrudate silica alumina support. These values represent an improvement in activity by 8.2% when powder silica alumina support is employed instead of extrudate silica alumina. The product yields obtained using the latter were 32.5, 25.2 and 9.4% after 5 h for *n*-olefins, monoesters and diesters compared to 26.0, 27.8 and 18.4% after 3 h, in that order, with a powdered support. Clearly there was an improvement in mono- and diester yields by 10.3 and 95.7%, respectively. On the other hand, the yield for *n*-olefins decreased by 20%. Indeed the results obtained and the trends observed in Sections 3.1–3.3 show a strong agreement and demonstrate a profound influence of particle size of the support on the activity and selectivity of the Re_2_O_7_ catalyst.

## 4. Conclusions

Although a 3 wt% Re_2_O_7_/SiO_2_-Al_2_O_3_/SnBu_4_ is already active for the metathesis of unsaturated FAMEs, the results have shown that particle size of silica alumina support has a profound influence on its activity and selectivity. Relatively low substrate conversions and lower yields in mono- and diesters are obtained upon using extrudate silica alumina as the support material. On the contrary, higher conversions with improved product yields and reaction rates are obtained with powder silica alumina. Pretreatment of extrudate silica alumina with either 2 wt% PO_4_^3–^ or 2 wt% Cs^+^ ions could, however, bring about a slight improvement in its catalytic activity. The low surface area and diffusion constraints in extrudates were identified as some of the factors leading to poor catalytic performance. Such constraints are minimal on silica alumina in powder form as a result of the high surface area of the support and the relatively easy contact between the substrate molecules and the catalytic active sites.

## Figures and Tables

**Table 1.** Yield (in mol%) of products resulting from self-metathesis of methyl linoleate with a 3% Re_2_O_7_/SiO_2_-Al_2_O_3_/SnBu_4_ catalytic system [substrate (0.5 mL), catalyst (0.2 g), temp (20 °C)].

Type of SiO_2_-Al_2_O_3_	Yield (%)				
	*n-*Olefins	Monoesters	Diesters	Total metathesis	Time/h
Extrudates	28.5	24.1	16.0	68.6	4
Powder	28.1	37.5	20.4	86.1	2

**Table 2.** Yield (in mol%) of products resulting from cross-metathesis of methyl oleate (MO) with methyl linoleate (ML) using a 3 wt% Re_2_O_7_/SiO_2_-Al_2_O_3_/SnBu_4_ catalytic system.

Type of SiO_2_-Al_2_O_3_	Yield (%)				
	*n-*Olefins	Monoesters	Diesters	Total metathesis	Time/h
Extrudates	38.0	22.8	6.0	66.8	5
Powder	23.4	33.4	19.4	76.2	3

ML/MO molar ratio = 3/1, substrate (0.5 mL), catalyst (0.2g), temp (20 °C)

**Table 3.** Yield (in mol%) of products resulting from metathesis of FAMEs (derived from sunflower oil) using 3 wt% Re_2_O_7_/SiO_2_-Al_2_O_3_/SnBu_4_ catalytic system [substrate (0.5 mL), catalyst (0.2g), temp (20 °C)].

Type of SiO_2_-Al_2_O_3_	Yield (%)				
	*n-*Olefins	Monoesters	Diesters	Total metathesis	Time/h
Extrudates	32.5	25.2	9.4	66.7	5
Powder	26.0	27.8	18.4	72.2	3
